# A New Up-conversion Material of Ho^3+^-Yb^3+^-Mg^2+^ Tri-doped TiO_2_ and Its Applications to Perovskite Solar Cells

**DOI:** 10.1186/s11671-018-2681-4

**Published:** 2018-08-31

**Authors:** Zhenlong Zhang, Danna Li, Wenjia Shi, Yanyan Liu, Yan Zhang, Yuefeng Liu, Huiping Gao, Yanli Mao

**Affiliations:** 1 School of Physics and Electronics, Henan University, Kaifeng, 475004 China; 2 Institute of Micro/Nano Photonic Materials and Applications, Henan University, Kaifeng, China; 3 Henan Vocational College of Applied Technology, Zhengzhou, 450042 China

**Keywords:** Ho^3+^-Yb^3+^-Mg^2+^ tri-doped TiO_2_, Up-conversion nanomaterial, Perovskite solar cells

## Abstract

A new up-conversion nanomaterial of Ho^3+^-Yb^3+^-Mg^2+^ tri-doped TiO_2_ (UC-Mg-TiO_2_) was designed and synthesized with a sol-gel method. The UC-Mg-TiO_2_ presented enhanced up-conversion fluorescence by an addition of Mg^2+^. The UC-Mg-TiO_2_ was utilized to fabricate perovskite solar cells by forming a thin layer on the electron transfer layer. The results display that the power conversion efficiency of the solar cells based on the electron transfer layer with UC-Mg-TiO_2_ is improved to 16.3 from 15.2% for those without UC-Mg-TiO_2_. It is demonstrated that the synthesized UC-Mg-TiO_2_ can convert the near-infrared light to visible light that perovskite film can absorb to improve the power conversion efficiency of the devices.

**Electronic supplementary material:**

The online version of this article (10.1186/s11671-018-2681-4) contains supplementary material, which is available to authorized users.

## Background

More attentions have been paid to the perovskite solar cells (PSCs) in the field of solar cells [1–5]. The power conversion efficiency (PCE) of the PSCs has been exceeding 22% within a few years [6]. However, the perovskite materials usually absorb the visible light whose wavelength is less than 800 nm, and more than half of the solar energy is not be utilized, especially in the region of near-infrared (NIR). To solve the issues, one of the effective methods is to apply the up-conversion nanomaterial to perovskite solar cells by converting the NIR light to visible light that the perovskite can utilize [7–9]. The beta-phase sodium yttrium fluoride (β-NaYF_4_) is commonly used as the host lattice for rare earth ions to prepare the up-conversion materials. While the β-NaYF_4_-based up-conversion materials are insulator, which is not beneficial for the electron transfer [ETL] [10].

Titanium dioxide (TiO_2_) nanocrystal with anatase phase is commonly used as the electron transfer material in the perovskite solar cells due to its suitable energy band structure, low cost, and long stability [11–13]. However, the energy band gap of TiO_2_ is large (3.2 eV), which hampers its applications. To improve the applications of TiO_2_ in visible light and near-infrared region, some methods were explored. One of the effective methods is doping TiO_2_ with metal or non-metal [14–16]. Yu et al. [17] demonstrated that Ho^3+^-Yb^3+^-F^−^ doped TiO_2_ could convert NIR light to visible light that can be absorbed by the dye-sensitized solar cells (DSSCs). Zhang and co-authors [18] proved that Mg-doped TiO_2_ can change the Fermi energy level of TiO_2_ to enhance the performance of perovskite solar cells.

In this work, we are preferred to combine the rear earth ions (Ho^3+^ and Yb^3+^) and the metal ion (Mg^2+^) doped TiO_2_ together to synthesize a new material with enhanced up-conversion fluorescence. Our purpose is to explore how the addition of Mg^2+^ affect the up-conversion fluorescence of TiO_2_ and to apply the up-conversion nanomaterial of Ho^3+^-Yb^3+^-Mg^2+^ tri-doped TiO_2_ to perovskite solar cells. The results display that the addition of Mg^2+^ enhanced the up-conversion emission of TiO_2_, and the application of Ho^3+^-Yb^3+^-Mg^2+^ tri-doped TiO_2_ improved the PCE of PSCs to 16.3% from 15.2%.

## Methods/Experimental

### Materials

Formamidinium iodide (FAI), Methylamium bromide (MABr), Lead diiodide (PbI_2_), 2,2′,7,7′-Tetrakis-(N,N-di-p-methoxyphenylamine)-9,9′-spirobifluorene (Spiro-OMeTAD), and lead dibromide (PbBr_2_) were purchased from Xi’an Polymer Light Technology Corp. (China). The SnO_2_ colloid solution was purchased from Alfa Aesar (tin (IV) oxide). Dimethyl sulfoxide (DMSO), N,N-dimethylformamide (DMF), 4-tert-butylpyridine (TBP), and lithium bis (trifluoromethanesulfonyl) imide (Li-TFSI) were purchased from Shanghai Aladdin Bio-Chem Technology Co., LTD (China).

### Synthesis of Ho^3+^-Yb^3+^-Mg^2+^ Tri-doped TiO_2_

The up-conversion material of Ho^3+^-Yb^3+^-Mg^2+^ tri-doped TiO_2_ was synthesized with a reported method [19] with some modifications. Firstly, a Titanium tetrabutanolate was obtained by mixing acetylacetone (AcAc) and Titanium tetrabutanolate (Ti(OBu)_4_) for 1 h under stirring at 25 °C, and then the isopropyl alcohol (IPA) was added to prepare the (Ti(OBu)_4_) solution. A mixed solution of IPA, HNO_3_, and H_2_O was dropped into the solutions slowly. After stirring for 6 h, a TiO_2_ sol with a color of light yellow was obtained. In a typical synthesis, the molar ratio of AcAc, HNO_3_, and H_2_O to Ti(OBu)_4_ was 1:0.3:2:1. For the synthesis of Ho^3+^-Yb^3+^ co-doped TiO_2_, Ho(NO_3_)_3_·5H_2_O and Yb(NO_3_)_3_·5H_2_O were used as the elemental sources and added into the solution. Typically, the molar ratio of Ho^3+^:Yb^3+^:Ti = 1:*x*:100 (*x* = 2, 3, 4, 5). For the synthesis of Ho^3+^-Yb^3+^-Mg^2+^ tri-doped TiO_2_, Ho(NO_3_)_3_·5H_2_O, Yb(NO_3_)_3_·5H_2_O, and Mg(NO_3_)_2_ 6H_2_O as the elemental sources were added into the solution, and the molar ratio of Ho^3+^:Yb^3+^:Mg^2+^:Ti = 1:4:*x*:100 (*x* = 0, 1, 1.5, 2, 2.5). The obtained solution was referred to as Ho^3+^-Yb^3+^-Mg^2+^ tri-doped TiO_2_ (UC-Mg-TiO_2_) sol. The solvent in the solution was removed by heating at 100 °C for 10 h. Then, the material powders were heated for 30 min at 500 °C.

### Preparation of PSCs

The FTO was washed in detergent, acetone, and isopropanol, and then treated for 15 min with UV-O_3_. A blocking layer was prepared by a spin-coating method using a solution of titanium diisopropoxide bis (acetylacetonate) in 1-butanol with the concentration of 1 M and then heated for 30 min at 500 °C. An electron transfer layer (ETL) prepared by a spin-coating method using TiO_2_ solution which is obtained by diluting TiO_2_ (30NR-D) using ethanol (1:6, mass ratio), and then heated for 10 min at 100 °C and 30 min at 450 °C. The UC-Mg-TiO_2_ was used to fabricate the solar cells by spin-coating a mixed solution of UC-Mg-TiO_2_ sol and TiO_2_ sol (UC-Mg-TiO_2_:TiO_2_ = *x*:(100 − *x*), *v*/*v*, *x* = 0, 20, 40, 60, 80, and 100) on the ETL and heating for 30 min at 500 °C. A perovskite film was fabricated according to the reported method [20]. In brief, the precursor solution of perovskite was prepared by dissolving FAI (1 M), PbI_2_ (1.1 M), MABr (0.2 M), and PbBr_2_ (0.22 M) in the mixture of DMF/DMSO (4:1 *v:v*), and a stock solution of CsI (1.5 M) in DMSO was added. The perovskite film was obtained by spin-coating method with 1000 rpm for 10 s and 4000 rpm for 30 s, and 200 μL chlorobenzene was dropped on the sample before the end of 20 s. A hole transfer layer (HTL) was obtained by the spin-coating method using a spiro-MeOTAD solution at 4000 rpm for 30 s. The spiro-OMeTAD solution was prepared by dissolving 72.3 mg spiro-MeOTAD in 1 mL chlorobenzene and by adding 28.8 μL TBP, 17.5 μL Li-TFSI solution (520 mg/ml in acetonitrile). Finally, an Au anode was made on the hole transfer layer by thermal evaporation.

### Characterization

Photoluminescence (PL) spectra were acquired using a fluorometer of FLS 980 E. A diffractometer of DX-2700 was used to obtain the X-ray diffraction (XRD) patterns. X-ray photoelectron spectra were measured with a spectrometer of XPS THS-103. Absorption spectra were obtained with a spectrophotometer of Varian Cary 5000. Scanning electron microscope (SEM) images were performed using a microscope of JSM-7001F. A Keithley 2440 Sourcemeter was applied to measure the photocurrent-voltage (I-V) curves of the solar cells under an illumination of AM 1.5. An electrochemical workstation of CHI660e was utilized to get the electrochemical impedance spectroscopy (EIS). The incident photon-to-current conversion efficiency (IPCE) was measured with a solar cell IPCE recording system (Crowntech Qtest Station 500ADX).

## Results and Discussion

**Fig. 1:**
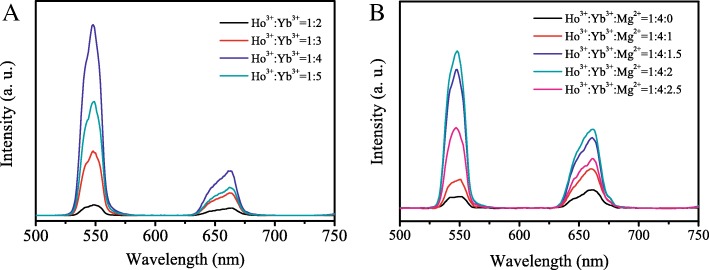
Up-conversion emissions of TiO_2_. **a** Ho^3+^-Yb^3+^ co-doped TiO_2_ (Ho:Yb:Ti = 1:*x*:100, molar ratio). **b** Ho^3+^-Yb^3+^-Mg^2+^ tri-doped TiO_2_ (Ho:Yb:Mg:Ti = 1:4:*x*:100, molar ratio)

The up-conversion fluorescence of the materials was optimized by varying the molar ratio of Ho^3+^ and Yb^3+^. The up-conversion emission of Ho^3+^-Yb^3+^ co-doped TiO_2_ with varying molar ratio of Ho^3+^ and Yb^3+^ (Ho:Yb:Ti = 1:*x*:100) was shown in Fig. 1a, which were excited with an 980 nm NIR light. Two strong up-conversion emission peaks were observed at 547 nm and 663 nm. Additional file 1: Figure S1 shows the up-conversion mechanisms of the Ho^3+^-Yb^3+^ co-doped TiO_2_. The fluorescence peaks at 663 nm and 547 nm could correspond to the ^5^F_5_ → ^5^I_8_ and (^5^S_2_, ^5^F_4_) → ^5^I_8_ transitions of Ho^3+^, respectively [21]. It can be seen that the intensity of the up-conversion fluorescence is the largest when the molar ratio of Ho^3+^ and Yb^3+^ is 1:4. Figure 1b presents the up-conversion photofluorescence of Ho^3+^-Yb^3+^-Mg^2+^ tri-doped TiO_2_ with different doping contents of Mg^2+^ (Ho:Yb:Mg:Ti = 1:4:*x*:100, molar ratio). The up-conversion fluorescence was enhanced by the addition of Mg^2+^. When the doping content of Ho^3+^:Yb^3+^:Mg^2+^ = 1:4:2, the up-conversion emission is the strongest for Ho^3+^-Yb^3+^-Mg^2+^ tri-doped TiO_2_. Hereinafter, the UC-Mg-TiO_2_ with the molar ratio of Ho^3+^:Yb^3+^:Mg^2+^:Ti = 1:4:2:100 was applied.

**Fig. 2:**
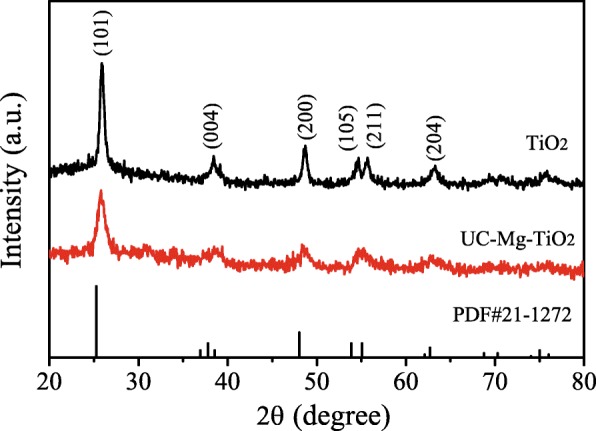
X-ray diffraction of TiO_2_ (30NR-D) and UC-Mg-TiO_2_

Figure 2 shows the X-ray diffraction of TiO_2_ (30NR-D) and UC-Mg-TiO_2_. According to the PDF card (JCPDS card no.21–1272), the peaks located at 2θ = 25.6 °, 37.7 °, 48.1 °, and 53.7 ° in the patterns could belong to the (101), (004), (200), (105), (211), and (204) crystal planes, respectively. This displays the phase of UC-Mg-TiO_2_ is anatase.

**Fig. 3:**
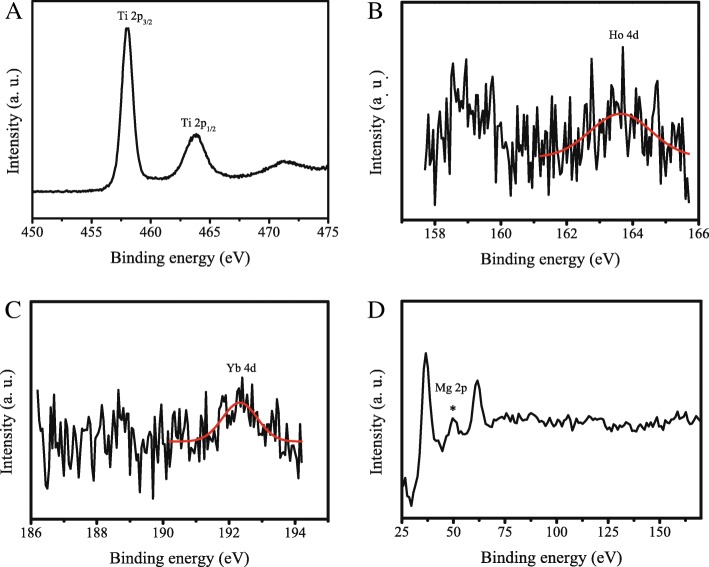
X-ray photoelectron spectra of UC-Mg-TiO_2_. **a** Ti 2p, **b** Ho 4d, **c** Yb 4d, and **d** Mg 2p

To demonstrate the doping of Ho, Yb, and Mg into TiO_2_, the X-ray photoelectron spectra of UC-Mg-TiO_2_ were obtained. The XPS survey spectrum of UC-Mg-TiO_2_ was presented in Additional file 1: Figure S2. Figure 3a shows the high-resolution photoelectron peaks of Ti 2p, which had two peaks of Ti 2p_1/2_ and Ti 2p_3/2_ located at 463.7 eV and 458.2 eV, respectively. Figure 3b, c shows the high-resolution photoelectron peaks of Ho 4d and Yb 4d, which appear at 163.6 eV and 192.3 eV, respectively. These agree with the reported peak positions [22]. Figure 3d presents the photoelectron peak of Mg 2p located at 49.8 eV [23]. These data displays that Ho, Yb, and Mg atoms were incorporated into TiO_2_.

**Fig. 4:**
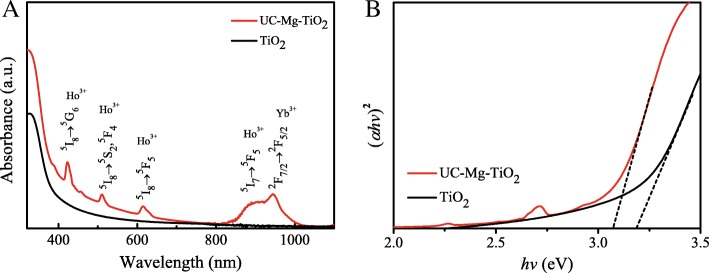
**a** Absorption spectra of TiO_2_ (30NR-D) and UC-Mg-TiO_2_. **b** Tauc plots

Figure 4a shows the absorption spectra of TiO_2_ (30NR-D) and UC-Mg-TiO_2_. There are five absorption peaks appear in the absorption spectrum of UC-Mg-TiO_2_, which are corresponding to characteristic absorption of Ho^3+^ and Yb^3+^. It can be seen that the doping of Ho, Yb, and Mg improves the absorption of TiO_2_ in visible light region and expands its absorption to NIR range. The Tauc plot can be used to estimate the energy band gap of material [24]. The Tauc plots from the absorption spectra were presented in Fig. 4b. The energy band gap values can be calculated to be 3.09 eV and 3.18 eV for UC-Mg-TiO_2_ and TiO_2_ (30NR-D), respectively. The UC-Mg-TiO_2_ presents a smaller band gap than TiO_2_.

**Fig. 5:**
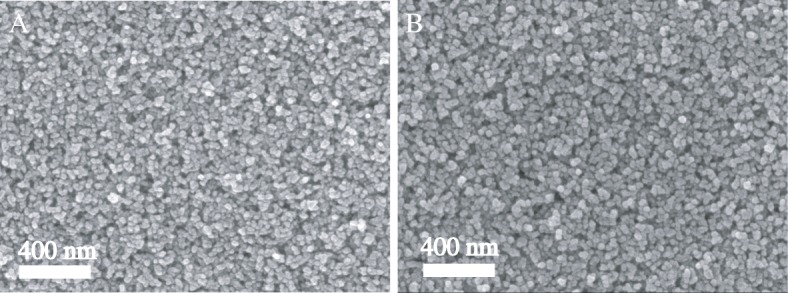
SEM photographs. **a** TiO_2_ (30NR-D) film. **b** UC-Mg-TiO_2_ film

Figure 5 shows the SEM photograph of TiO_2_ (30NR-D) and UC-Mg-TiO_2_ films. The size of the nanoparticle is about 25 nm for 30 NR-D, and particle size is about 28 nm for UC-Mg-TiO_2_. The two films are uniform. Thus, the UC-Mg-TiO_2_ displays a similar morphology and particle size to TiO_2_ (30NR-D).

**Fig. 6:**
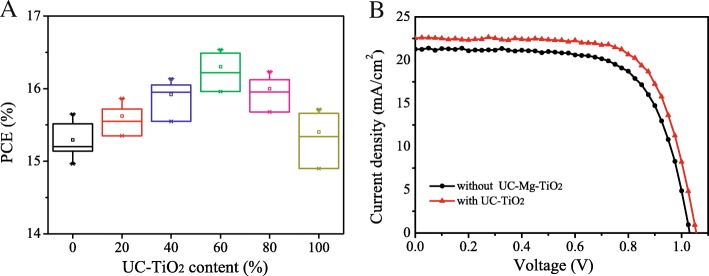
**a** Relationship between the PCE of devices and the contents of UC-Mg-TiO_2_ (UC-Mg-TiO_2_ sol: TiO_2_ sol = *x*:100 − *x*, *v*/*v*) in the mixed solution. **b** Typical I-V curves

The PSCs were fabricated based on the electron transfer layers with and without UC-Mg-TiO_2_. The electron transfer layer with UC-Mg-TiO_2_ was prepared by spin-coating the mixed solution of UC-Mg-TiO_2_ sol and TiO_2_ sol (UC-Mg-TiO_2_:TiO_2_ = *x*:(100 − *x*), *x* = 0, 20, 40, 60, 80, and 100, *v*/*v*). I-V measurements of the solar cells were performed, and from which the photovoltaic parameters were abstracted. The *I*_sc_, *V*_oc_, FF, and PCE of the solar cells in this work were obtained by an average of the values of 20 samples. The relation of PCE with the contents of UC-Mg-TiO_2_ was displayed in Fig. 6a. Firstly, the PCE of the solar cells becomes large, and after that becomes small with the increase of the UC-Mg-TiO_2_ contents, which reaches the maximum value at the content of 60% (UC-Mg-TiO_2_:TiO_2_ = 60:40, *v/v*). Table 1 presents the photovoltaic parameters of solar cells based on the electron transfer layers with and without UC-Mg-TiO_2_. The open-circuit voltage (*V*_oc_) and short-circuit current (*I*_sc_) of the solar cells with UC-Mg-TiO_2_ were increased to 1.05 V and 22.6 mA/cm^2^ from 1.03 V and 21.2 mA/cm^2^ for the solar cells without UC-Mg-TiO_2_, respectively. Thus, the PCE of the devices based on the electron transfer layer with UC-Mg-TiO_2_ was improved to 16.3% from 15.2% for those without UC-Mg-TiO_2_. The typical I-V curves of the devices are shown in Fig. 6b. The PCE histograms of the solar cell performance of 20 samples with and without UC-Mg-TiO_2_ are presented in Additional file 1: Figure S3.

**Table 1:** Photovoltaic parameters of the solar cells based on the mesoporous layers with and without UC-Mg-TiO_2_

Solar cells	*V*_oc_ (V)	*I*_sc_ (mA/cm^2^)	FF (%)	PCE (%)
Without UC-Mg-TiO_2_	1.03 ± 0.04	21.2 ± 0.7	69.6 ± 1.2	15.2 ± 0.5
With UC-Mg-TiO_2_	1.05 ± 0.03	22.6 ± 0.6	68.7 ± 1.3	16.3 ± 0.3

**Fig. 7:**
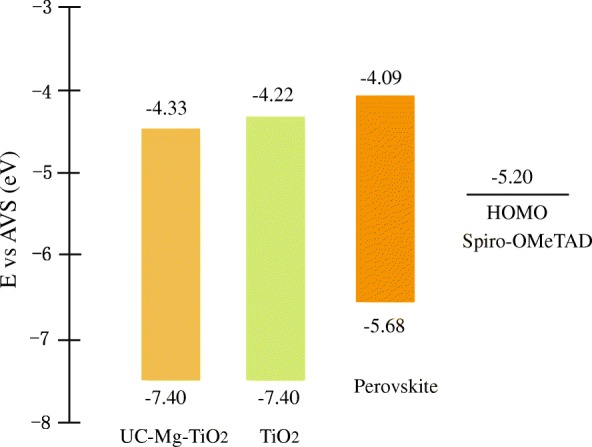
Energy band structures of the materials contained in the solar cells

Some experiments were carried out to explain the improvement. Figure 7 displays the energy band structures of the materials contained in the solar cells based on some reports [25, 26], and the energy band gap from the Tauc plots is shown in Fig. 4b. The conduction band difference between perovskite and TiO_2_ becomes larger for UC-Mg-TiO_2_ compared with that of TiO_2_ (30NR-D), since the UC-Mg-TiO_2_ has a smaller band gap than TiO_2_ (30NR-D). This may be one of the reasons to give a larger *V*_oc_ for the devices based on the electron transfer layer with UC-Mg-TiO_2_ [27, 28].

**Fig. 8:**
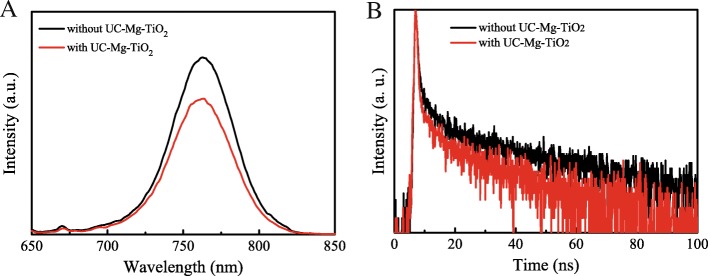
**a** Photoluminescence. **b** Time-resolved photoluminescence of perovskite film on TiO_2_ (30NR-D) and UC-Mg-TiO_2_

Figure 8a shows the steady-state photoluminescence (PL) of the perovskite films on the electron transfer layers with and without UC-Mg-TiO_2_. The PL peak located at 760 nm is originated from the perovskite film [29]. The PL intensity of the perovskite film on electron transfer layer with UC-Mg-TiO_2_ decreased compared with that of perovskite film on electron transfer layer without UC-Mg-TiO_2_. This implies that the electron transport and extraction of UC-Mg-TiO_2_ from the perovskite film is more efficient than that of TiO_2_ (30NR-D). This can be further demonstrated by the time-resolved photoluminescence (TRPL) of the samples shown in Fig. 8b. It can be seen that the decay time of TRPL for the perovskite film on electron transfer layer with UC-Mg-TiO_2_ is faster than that of perovskite film on electron transfer layer without UC-Mg-TiO_2_. This indicates that the charge transfer for the former is faster than the latter [30, 31].

**Fig. 9:**
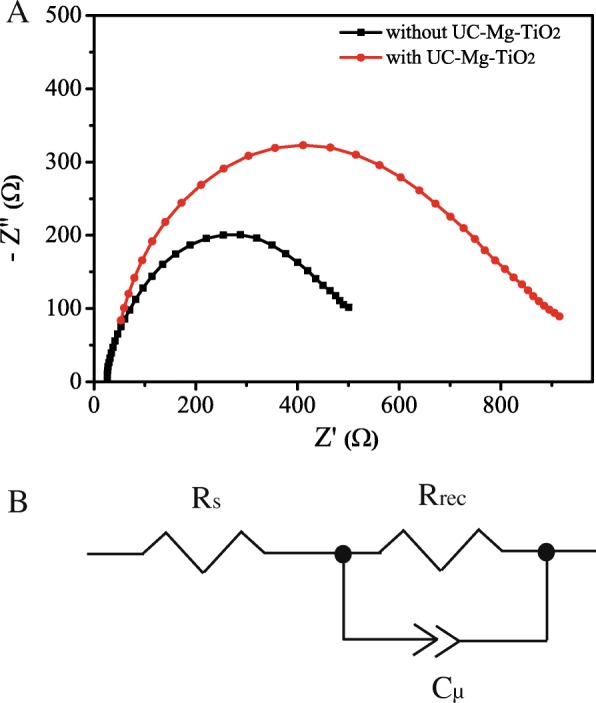
**a** Nyquist plots obtained from the EIS spectra. **b** Equivalent circuit utilized to analyze the EIS

Figure 9a shows the Nyquist plots obtained from the electrochemical impedance spectroscopy (EIS) of the solar cells based on the electron transfer layer with and without UC-Mg-TiO_2_. The Nyquist plots can be fitted by an equivalent circuit which is schematically shown in Fig. 9b. The *R*_s_, *R*_rec_, and *C*_μ_ are the series resistance, recombination resistance, and the capacitance of the device [32, 33]. The detailed fitting values are presented in Table 2. The *R*_s_ value of the devices based on the electron transfer layers with UC-Mg-TiO_2_ is nearly the same with that of those without UC-Mg-TiO_2_. While the *R*_rec_ value of the devices based on electron transfer layer with UC-Mg-TiO_2_ is larger than that of those without UC-Mg-TiO_2_. This implies that UC-Mg-TiO_2_ could effectively decrease the change recombination.

**Table 2:** Fitting parameters for EIS of the devices based on the electron transfer layer with and without UC-Mg-TiO_2_

Solar cells	*R*_s_/Ω	*R*_rec_/Ω	C_μ_-T/F	C_μ_-P
With UC-Mg-TiO_2_	23.4	489.2	9.9E-8	0.8
Without UC-Mg-TiO_2_	23.1	837.5	13.1E-8	0.8

**Fig. 10:**
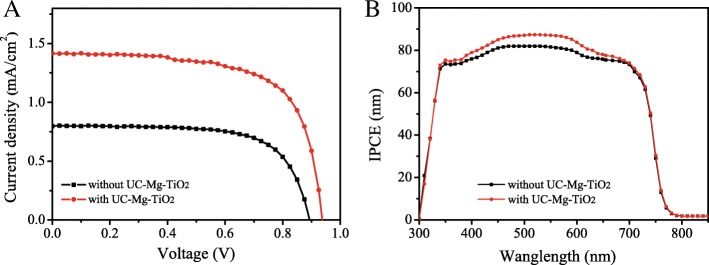
**a** I-V curves of the solar cells under the simulated solar radiation filtered with a band-pass NIR filter (980 ± 10 nm). **b** IPCE spectra of the solar cells with and without UC-Mg-TiO_2_

To confirm the contributions of the up-conversion material UC-Mg-TiO_2_ to the photocurrent of the solar cells, the I-V measurements were carried out under the simulated solar radiation filtered with a band-pass NIR filter (980 ± 10 nm). Figure 10a displays the I-V curves of the solar cells based on the electron transfer layers with and without UC-Mg-TiO_2_. The short-circuit current (*I*_sc_) of the solar cells with UC-Mg-TiO_2_ is obviously larger than that of those without UC-Mg-TiO_2_. This demonstrates the effect of UC-Mg-TiO_2_ on the photocurrent of the solar cells, because UC-Mg-TiO_2_ converts the near-infrared photons into visible photons, which the solar cells can absorb to produce additional photocurrent [7, 17]. Figure 10b shows the IPCE spectra of the solar cells with and without UC-Mg-TiO_2_. The IPCE of the solar cells with UC-Mg-TiO_2_ is increased, especially at the range of 400~650 nm, compared with that of those without UC-Mg-TiO_2_. This could be caused by the up-conversion effect of UC-Mg-TiO_2_ [7, 17].

## Conclusions

The up-conversion nanomaterial of Ho^3+^-Yb^3+^-Mg^2+^ tri-doped TiO_2_ (UC-Mg-TiO_2_) was synthesized successfully. The up-conversion emissions of the UC-Mg-TiO_2_ were enhanced with an addition of Mg^2+^. We applied the UC-Mg-TiO_2_ to the PSCs, in which the UC-Mg-TiO_2_ was used to modify the electron transfer layer. The *V*_oc_ and *I*_sc_ of the devices with UC-Mg-TiO_2_ were improved to 1.05 V and 22.6 mA/cm^2^ from 1.03 V and 21.2 mA/cm^2^ for those without UC-Mg-TiO_2_, respectively. And the PCE of the devices with UC-Mg-TiO_2_ was increased to 16.3% from 15.2% for those without UC-Mg-TiO_2_.

## Additional file

Additional file 1: **Figure S1.** Up-conversion mechanisms of the Ho^3+^-Yb^3+^ co-doped TiO_2_. **Figure S2.** XPS survey of UC TiO_2_. Figure S3 PCE histograms of the solar cell performance of 20 samples with and without UC-Mg-TiO_2_. (DOCX 77 kb)

## Abbreviations

EIS: Electrochemical impedance spectroscopy
NIR: Near-infrared
PCE: Power conversion efficiency
PL: Photoluminescence
PSCs: Perovskite solar cells
TRPL: Time-resolved photoluminescence

## References

[1] Yang WS, Noh JH, Jeon NJ, Kim YC, Ryu S, Seo J, Seok SI (2015). High-performance photovoltaic perovskite layers fabricated through intramolecular exchange. Science.

[2] Wang YF, Wu J, Zhang P, Liu DT, Zhang T, Ji L, Gu XL, Chen ZD, Li SB (2017). Stitching triple cation perovskite by a mixed anti-solvent process for high performance perovskite solar cells. Nano Energy.

[3] Li SB, Zhang P, Wang YF, Sarvari H, Liu DT, Wu J, Yang YJ, Wang ZM, Chen ZD (2017). Interface engineering of high efficiency perovskite solar cells based on ZnO nanorods using atomic layer deposition. Nano Res.

[4] Yang WS, Park BW, Jung EH, Jeon NJ, Kim YC, Lee DC, Shin SS, Seo J, Kim EK, Noh JH, Seok SI (2017). Iodide management in formamidinium lead halide based perovskite layers for efficient solar cells. Science.

[5] Zhang T, Wu J, Zhang P, Ahmad W, Wang YF, Alqahtani M, Chen H, Gao CM, Chen ZD, Wang ZM, Li SB (2018). High speed and stable solution-processed triple cation perovskite photodetectors. Adv Optical Mater.

[6] Jeon NJ, Na H, Jung EH, Yang TY, Lee YG, Kim G, Shin HW, Seok SI, Lee J (2018) A fluorene-terminated hole-transporting material for highly efficient and stable perovskite solar cells. Nature Energy. 10.1038/s41560-018-0200-6

[7] He M, Pang XC, Liu XQ, Jiang BB, He YJ, Snaith H, Lin ZQ (2016). Monodisperse dual-functional upconversion nanoparticles enabled near-infrared organolead halide perovskite solar cells. Angew Chem Int Ed.

[8] Roh JM, Yu HJ, Jang J (2016). Hexagonal β-NaYF_4_:Yb^3+^, Er^3+^ nanoprism incorporated upconverting layer in perovskite solar cells for near-infrared sunlight harvesting. ACS Appl Mater Interfaces.

[9] Zhang P, Li SB, Liu CH, Wei XB, Wu ZM, Jiang YD, Chen Z (2014). Near-infrared optical absorption enhanced in black silicon via Ag nanoparticle-induced localized surface plasmon. Nanoscale Res Lett.

[10] Yu J, Yang YL, Fan RQ, Zhang HJ, Li L, Wei LG, Shi Y, Pan K, Fu HG (2013). Er^3+^ and Yb^3+^ co-doped TiO_2-x_F_x_ up-conversion luminescence powder as a light scattering layer with enhanced performance in dye sensitized solar cells. J Power Sources.

[11] You S, Wang H, Bi SQ, Zhou JY, Qin L, Qiu XH, Zhao ZQ, Xu Y, Zhang Y, Shi XH, Zhou HQ, Tang ZY (2018). A biopolymer heparin sodium interlayer anchoring TiO_2_ and MAPbI_3_ enhances trap passivation and device stability in perovskite solar cells. Adv Mater.

[12] Kim W, Park J, Kim H, Pak Y, Lee H, Jung GY (2017). Sequential dip-spin coating method: fully infiltration of MAPbI_3-x_Cl_x_ into mesoporous TiO_2_ for stable hybrid perovskite solar cells. Electrochemi Acta.

[13] Choi J, Song S, Horantner MT, Snaith HJ, Park T (2016). Well-defined nanostructured, single crystalline TiO_2_ electron transport layer for efficient planar perovskite solar cells. ASC Nano.

[14] Giordano F, Abate A, Baena JPC, Saliba M, Matsui T, Im SH, Zakeeruddin SM, Nazeeruddin MK, Hagfeldt A, Graetzel M (2016). Enhanced electronic properties in mesoporous TiO_2_ via lithium doping for high-efficiency perovskite solar cells. Nat Commun.

[15] Liu JW, Zhang J, Yue GQ, Lu XW, Hu ZY, Zhu YJ (2016). W-doped TiO_2_ photoanode for high performance perovskite solar cell. Electrochimi Acta.

[16] Wu MC, Chan SH, Jao MH, Su WF (2016). Enhanced short-circuit current density of perovskite solar cells using Zn-doped TiO_2_ as electron transport layer. Sol Energ Mater Sol C.

[17] Yu J, Yang Y, Fan R, Liu DQ, Wei LG, Chen S, Li L, Yang B, Cao WW (2014). Enhanced near-infrared to visible upconversion nanoparticles of Ho^3+^-Yb^3+^-F^−^ tri-doped TiO_2_ and its application in dye-sensitized solar cells with 37% improvement in power conversion efficiency. Inorg Chem.

[18] Zhang HY, Shi JJ, Xu X, Zhu LF, Luo YH, Li DM, Meng QB (2016). Mg-doped TiO_2_ boosts the efficiency of planar perovskite solar cells to exceed 19%. J Mater Chem A.

[19] He YY, Wu JL, Wang XH, Feng ZQ, Dong B (2016). Optical temperature sensing behavior through stark sublevels transitions of green and red upconversion emissions for Er^3+^-Yb^3+^-Li^+^ codoped TiO_2_ phosphors. J Nanosci Nanotechnol.

[20] Michael S, Taisuke M, Seo JY, Domanski K, Correa-Baena JP, Nazeeruddin MK, Zakeeruddin SM, Tress W, Abate A, Hagfeldtd A, Gratzel M (2016). Cesium-containing triple cation perovskite solar cells: improved stability, reproducibility and high efficiency. Energy Environ Sci.

[21] Huang X, Han S, Huang W, Liu X (2013). Enhancing solar cell efficiency: the search for luminescent materials as spectral converters. Chem Soc Rev.

[22] Mazierski P, Lisowski W, Grzyb T, Winiarski MJ, Klimczuk T, Mikolajczyk A, Flisikowski J, Hirsch A, Kolakowska A, Puzyn T, Zaleska-Medynska A, Nadolna J (2017). Enhanced photocatalytic properties of lanthanide-TiO_2_ nanotubes: an experimental and theoretical study. Appl Catalysis B.

[23] Cheng G, Akhtar MS, Yang OB, Stadler FJ (2016). Nanoprecursor-mediated synthesis of Mg^2+^-doped TiO_2_ nanoparticles and their application for dye-sensitized solar cells. J Nanosci Nanotechno.

[24] Gao XX, Ge QQ, Xue DJ, Ding J, Ma JY, Chen YX, Zhang B, Feng YQ, Wan LJ, Hu JS (2016). Tuning the fermi-level of TiO_2_ mesoporous layer by lanthanum doping towards efficient perovskite solar cells. Nanoscale.

[25] Chen H, Yang B, Shuang X, Zhang T, Meng XY, Ng WK, Yang YL, Wong KS, Chen HN, Yang SH (2017). Tuning the A-site cation composition of FA perovskites for efficient and stable NiO-based p-i-n perovskite solar cells. J Mater Chem A.

[26] Rhee JH, Chung CC, Diau WG (2013). A perspective of mesoscopic solar cells based on metal chalcogenide quantum dots and organometal-halide perovskites. NPG Asia Mater.

[27] Li YL, Sun WH, Yan WB, Ye SY, Peng HT, Liu ZW, Bian ZQ, Huang CH (2015). High-performance planar solar cells based on CH_3_NH_3_PbI_3-x_Cl_x_ perovskites with determined chlorine mole fraction. Adv Funct Mater.

[28] Kim HS, Park NG (2014). Parameters affecting I-V hysteresis of CH_3_NH_3_PbI_3_ perovskite solar cells: effects of perovskite crystal size and mesoporous TiO_2_ layer. J Phys Chem Lett.

[29] Grancini G, Roldán-Carmona C, Zimmermann I, Mosconi E, Lee X, Martineau D, Narbey S, Oswald F, De Angelis F, Graetzel M, Nazeeruddina MK (2017). One-year stable perovskite solar cells by 2D/3D interface engineering. Nat Commun.

[30] Zhao L, Luo D, Wu J, Hu Q, Zhang W, Chen K, Liu T, Liu Y, Zhang Y, Liu F, Russell TP, Snaith HJ, Zhu R, Gong Q (2016). High-performance inverted planar heterojunction perovskite solar cells based on lead acetate precursor with efficiency exceeding 18. Adv Funct Mater.

[31] Zhou H, Chen Q, Li G, Luo S, Song TB, Duan HS, Hong Z, You J, Liu Y, Yang Y (2014). Interface engineering of highly efficient perovskite solar cells. Science.

[32] Huang Y, Zhu J, Ding Y, Chen S, Zhang C, Dai S (2016). TiO_2_ sub-microsphere film as scaffold layer for efficient perovskite solar cells. ACS Appl Mater Inter.

[33] Yun J, Ryu J, Lee J, Yu H, Jang J (2016). SiO_2_/TiO_2_ based hollow nanostructures as scaffold layers and Al-doping in the electron transfer layer for efficient perovskite solar cells. J Mater Chem A.

