# Supplementary material for: A New Up-conversion Material of Ho3+-Yb3+-Mg2+ Tri-doped TiO2 and Its Applications to Perovskite Solar Cells
Source: Nanoscale Res Lett. 2018 Aug 31;13:262. doi: 10.1186/s11671-018-2681-4 (PMC6119175; doi:10.1186/s11671-018-2681-4)
Supplement: Supplementary file 1 — Figure S1. Up-conversion mechanisms of the Ho3+-Yb3+ co-doped TiO2. Figure S2. XPS survey of UC TiO2. Figure S3 PCE histograms of the solar cell performance of 20 samples with and without UC-Mg-TiO2. (DOCX 77 kb) [file 11671_2018_2681_MOESM1_ESM.docx]

**Additional file 1**

**A New Up-conversion Material of Ho^3+^-Yb^3+^-Mg^2+^ Doped TiO_2_**

**And Its Applications to Perovskite Solar Cells**

Zhenlong Zhang ^a, b^, Danna Li ^c^, Wenjia Shi ^a^, Yanyan Liu ^a^, Yan Zhang ^a^,

Yuefeng Liu ^a^, Huiping Gao ^a^, Yanli Mao ^a,b,*^

^a^ School of Physics and Electronics, Henan University, Kaifeng 475004, China

^b^ Institute of Micro/Nano Photonic Materials and Applications, Henan University

^c^ Henan Vocational College of Applied Technology, Zhengzhou 450042, China


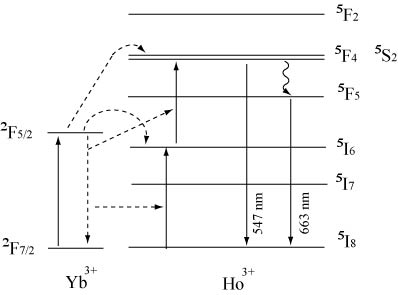


Figure S1 Up-conversion mechanisms of the Ho^3+^-Yb^3+^ co-doped TiO_2_.

Figure S2 XPS survey of UC TiO_2_.

Figure S3 PCE histograms of the solar cell performance of 20 samples with and without UC-Mg-TiO_2_.
